# Size Distribution of Air Bubbles Entering the Brain during Cardiac Surgery

**DOI:** 10.1371/journal.pone.0122166

**Published:** 2015-04-02

**Authors:** Emma M. L. Chung, Caroline Banahan, Nikil Patel, Justyna Janus, David Marshall, Mark A. Horsfield, Clément Rousseau, Jonathan Keelan, David H. Evans, James P. Hague

**Affiliations:** 1 University of Leicester Department of Cardiovascular Sciences, Leicester, United Kingdom; 2 University Hospitals of Leicester NHS Trust, Leicester, United Kingdom; 3 Leicester Cardiovascular Biomedical Research Unit, Leicester, United Kingdom; 4 Open University Department of Physical Sciences, Milton Keynes, United Kingdom; University of Washington, UNITED STATES

## Abstract

**Background:**

Thousands of air bubbles enter the cerebral circulation during cardiac surgery, but whether high numbers of bubbles explain post-operative cognitive decline is currently controversial. This study estimates the size distribution of air bubbles and volume of air entering the cerebral arteries intra-operatively based on analysis of transcranial Doppler ultrasound data.

**Methods:**

Transcranial Doppler ultrasound recordings from ten patients undergoing heart surgery were analysed for the presence of embolic signals. The backscattered intensity of each embolic signal was modelled based on ultrasound scattering theory to provide an estimate of bubble diameter. The impact of showers of bubbles on cerebral blood-flow was then investigated using patient-specific Monte-Carlo simulations to model the accumulation and clearance of bubbles within a model vasculature.

**Results:**

Analysis of Doppler ultrasound recordings revealed a minimum of 371 and maximum of 6476 bubbles entering the middle cerebral artery territories during surgery. This was estimated to correspond to a total volume of air ranging between 0.003 and 0.12 mL. Based on analysis of a total of 18667 embolic signals, the median diameter of bubbles entering the cerebral arteries was 33 μm (IQR: 18 to 69 μm). Although bubble diameters ranged from ~5 μm to 3.5 mm, the majority (85%) were less than 100 μm. Numerous small bubbles detected during cardiopulmonary bypass were estimated by Monte-Carlo simulation to be benign. However, during weaning from bypass, showers containing large macro-bubbles were observed, which were estimated to transiently affect up to 2.2% of arterioles.

**Conclusions:**

Detailed analysis of Doppler ultrasound data can be used to provide an estimate of bubble diameter, total volume of air, and the likely impact of embolic showers on cerebral blood flow. Although bubbles are alarmingly numerous during surgery, our simulations suggest that the majority of bubbles are too small to be harmful.

## Introduction

Air bubbles entering the cerebral circulation intra-operatively have potential to obstruct blood flow, and are thought to be a source of endothelial irritation and inflammation.[1,2] Although bubbles entering the circulation during heart surgery have long been implicated as a possible cause of post-operative cognitive decline, the potential for adverse clinical sequelae due to bubbles in the bloodstream remains controversial.[2–5] Potential alternative explanations for cognitive decline include the effects of age, pre-existing cognitive decline, and pre-existing cardiovascular disease, combined with peri-operative stressors, such as the use of anaesthesics, haemodynamic changes during cardiopulmonary bypass, impact of particulate emboli, impaired regulation of cerebral blood flow, and inflammatory responses. Although studies of decompression sickness[1] and experiments on animals[6] clearly demonstrate the potential for clinical symptoms if bubbles are present in sufficient quantities, interventional trials conducted in a cardiac surgery setting find no cognitive benefit in reducing the volume of air entering the bloodstream during surgery [3,7]. Since there is currently no method for determining the size distribution and volume of bubbles reaching the cerebral circulation, it is difficult to assess whether quantities of air typically introduced during surgery are high enough to result in cognitive decline.

Transcranial Doppler (TCD) ultrasound techniques are highly sensitive to small air emboli and typically reveal thousands of embolic signals originating from tiny air bubbles entering the brain during surgery. Since any potential adverse impact of bubbles on cerebral blood flow is sensitively related to bubble size, the ability to estimate the sizes of bubbles in the bloodstream may be useful for distinguishing clinically significant macrobubbles from tiny microbubbles that pass harmlessly through the vasculature and are outgassed by the lungs.[2] Although existing technologies are capable of detecting and sizing bubbles moving through Cardiopulmonary Bypass (CPB) lines,[8] no studies have so far been performed to investigate the size distribution and volume of air entering the brain intra-operatively.

The aim of this study was to estimate the size distribution and volume of air entering the Middle Cerebral Artery (MCA) territories of 10 patients during cardiac surgery. Bubble sizes were estimated based on analysis of transcranial Doppler (TCD) ultrasound embolic signals. We demonstrate that analysis of the backscattered ultrasound intensity of embolic signals can be used to estimate the sizes of bubbles, and total volume of air entering the MCA territory. These data were then input into a patient-specific Monte-Carlo simulation[9] of emboli moving through the vasculature to offer an initial estimate for the cumulative impact of bubbles on cerebrovascular perfusion. Overall, the aim of our study was to offer an estimate for the size distribution of bubbles, volume of air, and magnitude and time dependence of cerebrovascular obstruction associated with bubbles entering the bloodstream during cardiac surgery.

## Patients and Methods

All clinical data were collected in accordance with local research ethics procedures specifically approved for this study by the University of Leicester and UK National Health Research Ethics Service (NRES) Committee East Midlands—Derby, reference 10/H0401/78. All patients provided written informed consent for their anonymised data to be used in this research. We analysed TCD recordings from 10 patients monitored bilaterally during Coronary Artery Bypass Graft (CABG) and/or mitral or aortic valve surgery (MVR/AVR) requiring cardiopulmonary bypass (CPB). Data were collected as part of a larger study investigating brain injury following cardiac surgery funded by the British Heart Foundation.

Intra-operative TCD monitoring was performed using a commercially available TCD system (DWL Doppler Box, Compumedics, Germany) equipped with a pair of 2 MHz transducers. Probes were held in place using an adjustable head frame and settings were optimised to obtain a clear signal from the MCA. Stages of the surgery and fluctuations in blood pressure (BP) and haematocrit were noted in a detailed transcript and cross-referenced to the perfusionist's notes to match the timing of embolic signals with surgical and perfusionist interventions.

'In house' Doppler signal analysis software was developed in MATLAB (The Mathworks Inc., Natick, MA) to display all Doppler signals exceeding the background blood flow signal by at least 7 dB. Based on inspection of the sonogram and audio signal by a trained expert, each signal was either accepted as an embolus or rejected as an artefact according to internationally agreed consensus criteria.[10] The backscattered embolic signal intensity (I_E+B_) for each accepted signal was then estimated relative to the scattering from blood (I_B_) as a peak Measured Embolus-to-Blood Ratio (MEBR) in decibels (dB), Eq. 1:

$$\text{MEBR}=10{\text{log}}_{10}\left[\frac{I_{E+B}}{I_{B}}\right]\;\text{dB}$$ (1)

In sections of our recordings where embolisation rates exceeded 5 emboli/s it became difficult to identify a suitable background signal. Where this occurred a background signal was selected just prior to shower commencement and this value was then used to calculate MEBR values for all emboli within the shower. For higher rates of embolisation during dense embolic showers (‘curtains’ of emboli) it became impossible to distinguish individual emboli and the duration of the curtain was recorded.

Bubble sizes were estimated using an algorithm developed by Banahan *et al*. 2012.[11] This is based on theoretical models describing backscattered ultrasound from a spherical embolus moving through a blood-filled vessel.[12] In tests, 91% of 10,000 randomly generated simulated emboli were correctly sized to within 10% of their true dimensions.[11] The algorithm includes estimates for Doppler sample length (which varied between 8 and 12 mm), and Doppler angle (assumed to be 30°). As changes in haematocrit concentration can affect estimates of MEBR by up to 5 dB, backscattered intensities were adjusted for intra-operative haematocrit values based on the results of perfusionist blood sampling. Blood sampling was performed every 3 minutes during bypass. To ensure our bubble sizing algorithm was as accurate as possible, patient-specific measurements of MCA diameter were also obtained by 3D reconstruction of the circle of Willis of each patient using time-of-flight MR angiography (Magnetom Skyra, Siemens Medical, Erlangen, Germany).

The largest source of error in estimating bubble sizes originates from beam-vessel misalignment. *In-vitro* experiments found that good beam-vessel alignment lead to on average ~15% error in size which increased to a maximum of ~ 50% error for bubble diameters > 1mm.[11] In our calculations an error of 40% was assumed for all estimated diameters. Care was taken to optimise the blood signal from the MCA for each recording to obtain good beam-vessel alignment. Conversion of bubble diameters to volume of air using $V=\frac{4}{3}\pi r^{3}$ was used to estimate the total volume of air entering the MCA territories for each patient.

To quantify the impact of showers of emboli on cerebrovascular perfusion, the accumulation and clearance of air bubbles over time was modelled using a Monte-Carlo simulation simulating the motion of gaseous emboli through a bifurcating arterial tree.[9] The tree was chosen to mimic the topology of the MCA microvasculature with the root node adjusted to match the diameter of the MCA, determined for each patient using time-of-flight magnetic resonance angiography. The model vasculature comprised 20 layers of bifurcations terminating in ~500,000 terminal arterioles (~26 μm in diameter). This simulation incorporates the effects of bubble deformation, blood pressure, and friction between the surface of the bubble and the vessel wall.

The aim of developing this model was to make predictions of the state of the cerebral vascular tree when many emboli of different sizes are introduced, based on the underlying hypothesis that the paths and destinations of emboli can be determined probabilistically. Previous work has shown this approach to be reasonable, since the total number of obstructed arteries predicted by the model is not strongly dependent on the specific form of the probabilities that determine the paths of the emboli.[14] We start by simplifying the cerebral vasculature into a mathematical tree featuring symmetric bifurcations linked by arteries. Vessel diameters in the model vasculature follow Murray’s law, such that at each bifurcation the vessels become smaller by a factor of 2^1/3^. Initialisation occurs by calculating all flows and pressures in the tree assuming Poiseuille flow. Into this tree, we introduce emboli with radii taken from the sizing algorithm. These values are slightly modified with random noise each time the code is run such that the size distribution after many runs has a standard deviation equivalent to the sizing uncertainty. On each time-step emboli move into the tree until they reach the next bifurcation, where the direction that the embolus takes is determined probabilistically according to the relative flows in the two daughter vessels of the bifurcation. This continues until the emboli reach an artery where the limiting static friction (stiction) F = KA_wall_ matches the force generated by the pressure differential over the embolus (F = pA_X_) and therefore forms a blockage. The coefficient of stiction was taken from experimental values (K = 10 N m^-2^), A_wall_ is the area of the embolus that in contact with the wall, and A_x_ = πr^2^ is the cross sectional area of the vessel, where r is the radius of the vessel. At this point, a new flow distribution is calculated for the tree. On each time-step the proportion of terminal arterioles of the tree receiving flow is determined. In previous studies, buoyancy was found to only have a small effect on total predicted levels of obstruction, leading to a slight reduction in the predicted value.[14] Therefore, in the simulations conducted for this study buoyancy was neglected such that the estimated proportion of blocked arteries represent an upper limit on the actual number of blockages. In all calculations, the input pressure was assumed to be 100 mmHg.

A probabilistic (Monte Carlo) approach is necessary since it is impossible to make detailed fluid dynamical simulations in a tree with over 10^6^ components, especially when the initial positions of emboli within the cross section of the MCA is unknown. The Monte Carlo approach also helps with propagating uncertainties in the bubble sizing into our estimates. Multiple simulations are run to average over the uncertainties in the embolus sizing and also to ensure that the probabilistic approach to the directions that emboli take at bifurcations is also averaged. The approach is valid when there are large numbers of emboli, which is true in a cardiac surgery setting. The Monte Carlo aspect of averaging over ensembles means that the 95% Confidence Interval on each prediction can be calculated. The symmetrisation of the bifurcations and the neglect of buoyancy in the absence of detailed spatial information about the tree mean that the calculations presented here offer an upper estimate on the proportion of obstructed arteries. By modelling in this way, we overcome the problems of trying to adjust for embolus volume in weighting the impact of emboli on cerebrovascular perfusion. Bubble radii (including estimated uncertainties) and blood pressure were input to the model MCA vasculature as a function of time and each simulation run 30 times to determine the average number and duration of obstructed end arterioles during surgery. Uncertainty in the estimated instantaneous percentage of affected arterioles was quantified in the form of 95% Confidence Interval. Full details of our Monte-Carlo simulation are described in previous publications.[9,13,14]

## Results

Bilateral MCA monitoring of 10 patients during cardiac surgery yielded over 17 hours of TCD recordings containing 18667 individual embolic signals. A summary of parameters recorded during surgery, and the results of our analysis, are presented in Table 1.

**Table 1:** Summary of patient details, intra-operative haematocrit, and analysis of TCD recordings.

Patient number	1	2	3	4	5	6	7	8	9	10
Gender, (m/f)	M	m	m	m	m	m	f	m	m	m
Age, (years)	71	66	74	60	55	64	71	71	76	69
MCA^a^ diameter left:right, (mm)	2.6:2.7	2.6:2.8	2.9:2.6	2.6:2.9	3.5:2.6	3.3:3.2	3:3.1	2.6:2.8	2.9:2.8	2.6:2.7
Procedure	MVR^c^	CABG^d^ MVR^c^	CABG^d^ MVR^c^	CABG^d^ x3	CABG^d^ AVR^e^	MVR^c^	CABG^d^	CABG^d^ x3	CABG^d^ x3	CABG^d^ x2
Haematocrit on CPB^b^, % (range)	30 (30–33)	28 (26–32)	33 (28–36)	33 (32–34)	28 (27–33)	31 (29–35)	23 (22–30)	34 (30–37)	26 (22–30)	31 (29–34)
Number of emboli, left:right	502:596	349:322	2785:3691	409:383	2080:2144	480:886	674:843	196:175	950:601	284:317
Total volume (mL)	0.021	0.016	0.005	0.0003	0.12	0.003	0.04	0.0002	0.022	0.01
Signal Loss, left:right (mins)	0:6	5:0	0:0	5:11	0:0	12:0	0:4	4:10	0:0	0:0
Curtain duration, left:right (secs)	7:7	0:0	0:0	0:0	65:65	31:31	0:0	0:0	22:15	0:0
Affected arterioles, left:right (%)	0.38:0.30	0.41:0.15	0.1:0.11	0.007:0.005	1.01:2.25	0.04:0.03	0.15:0.79	0.002:0.007	0.46:0.22	0.006:0.034

^a^MCA—Middle Cerebral Artery

^b^CPB—Cardiopulmonary Bypass

^c^MVR—Mitral Valve Replacement

^d^CABG—Coronary Artery Bypass Graft

^e^AVR—Atrial Valve Replacement.

**Fig 1:**
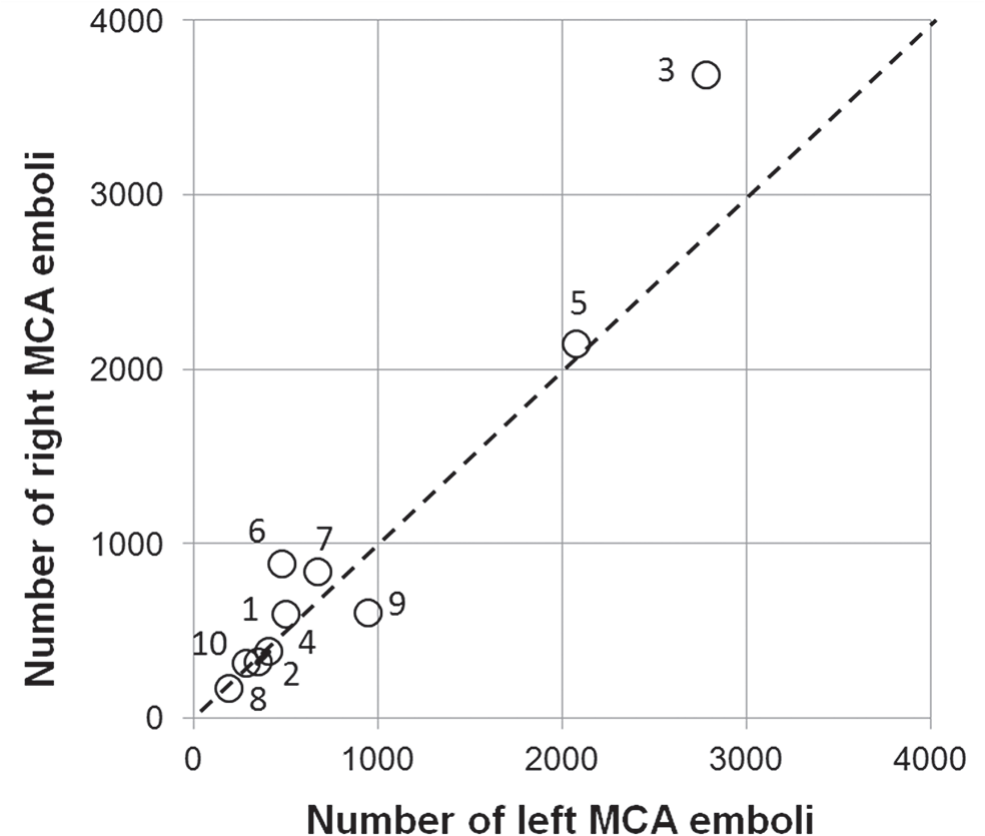
Total numbers of bubbles detected in the left and right middle cerebral arteries. Similar numbers of bubbles were detected in the left and right MCAs of individual patients. Markers are labelled with patient identifiers.

Total numbers of emboli entering the MCAs during a single operation varied from a minimum of 371 (patient 8) to a maximum of 6476 (patient 3). Similar total numbers of emboli were observed in the left and right MCAs, Fig 1. A paired t-test confirmed no significant difference in the median number of emboli in the left [491 (IQR: 349 to 950)] or the right sides [599 (IQR: 322 to 886)], (paired t-test, *t* = -1.19, *p* = 0.27). Higher average numbers of bubbles tended to be observed during the 5 intra-cardiac procedures (mean 1366, range: 671, 6476) than the 5 CABG procedures (mean 792, range: 371, 1551), though with so few patients this did not reach significance (Wilcoxon rank-sum test, *z* = -1.15, *p* = 0.25).

**Fig 2:**
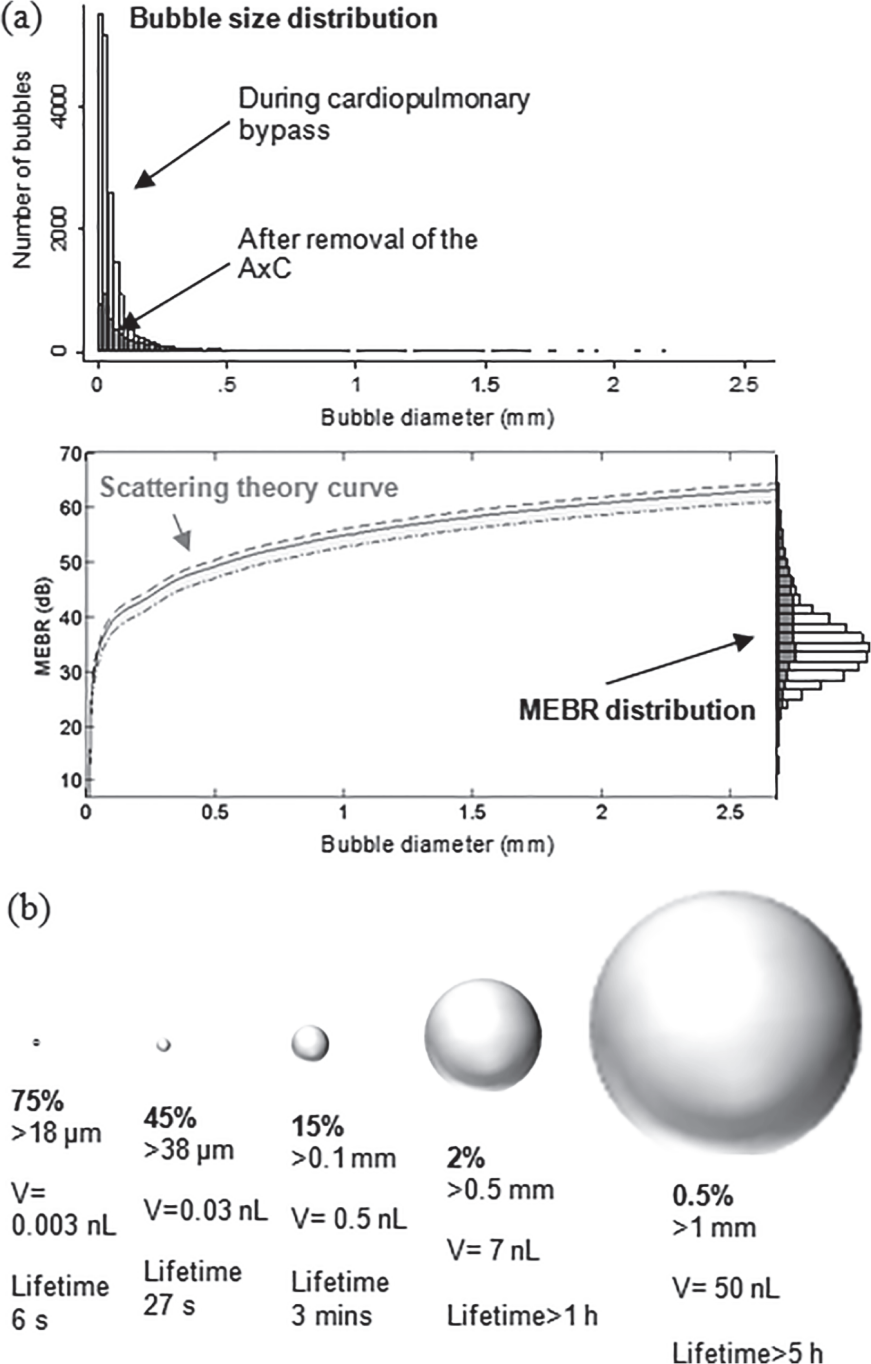
Estimated distribution of bubble sizes detected during cardiac surgery. (a) Distribution of bubble sizes estimated based on analysis of the ultrasound backscatter (MEBR values) from 18667 embolic signals. Overall, the median diameter of bubbles was 33 μm (IQR: 18 to 69 μm). Signals observed following removal of the aortic cross-clamp are shaded. (b) Percentage of emboli, bubble volume, and predicted dissolve times for 18 μm, 38 μm, 100 μm, 500 μm and 1 mm diameter air bubbles.

Patient-specific bubble sizing revealed that the majority (85%) of bubbles were less than 100 μm and can be expected to dissolve within minutes, Fig 2. The median bubble diameter from all 18667 signals was 33 μm (IQR: 18 to 71 μm). Conversion of MEBR values to bubble diameters is illustrated in Fig 2(A). Note that the exact form of this curve varies slightly between patients depending on MCA diameter and intra-operative haematocrit levels.[11] A summary of the percentage of bubbles above a particular diameter, and their estimated dissolve times, is provided in Fig 2(B). Only 2.2% of bubbles were larger than 500 μm. Of these, approximately 90 bubbles (0.5%) were estimated to represent clinically significant macrobubbles over 1 mm in diameter.

**Fig 3:**
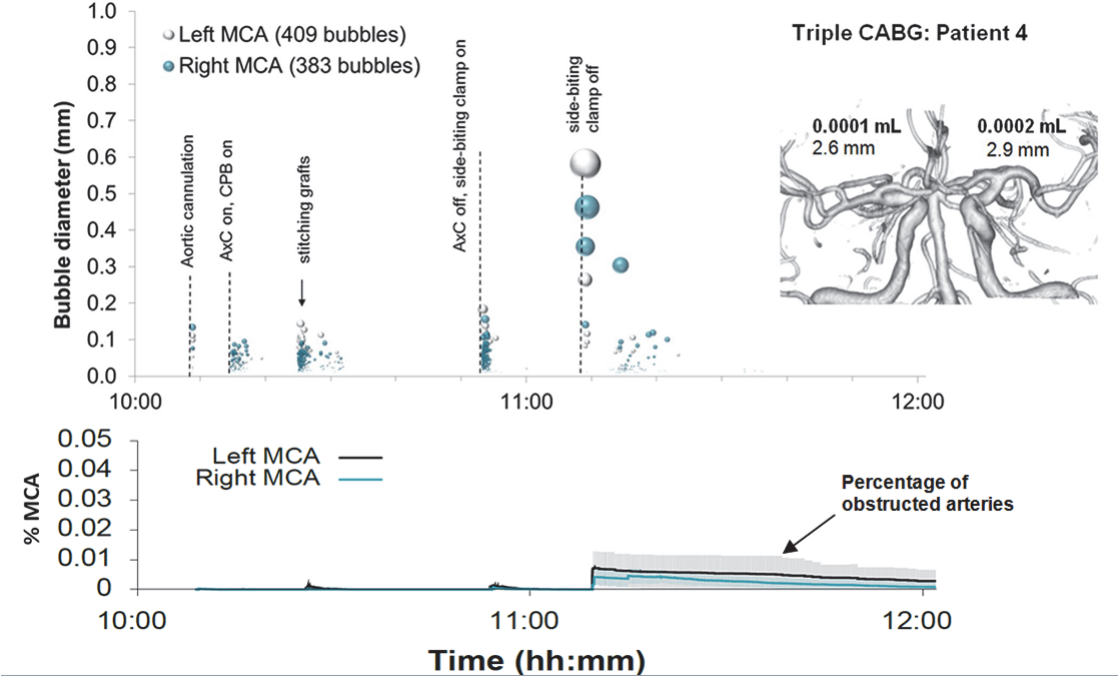
Timing and diameters of bubbles in a 60 year old patient during Triple CABG. Markers denote individual embolic events where the y-axis and marker size indicate estimated bubble diameter. The lower panel displays the predicted number of blocked MCA end arterioles estimated by Monte-Carlo simulation. The shaded regions highlight 95% confidence intervals, based on uncertainty in bubble diameter and variations in outcome between simulations. The inset shows a 3D reconstruction of the circle of Willis labelled with estimated total volume of air and MCA diameters.

**Fig 4:**
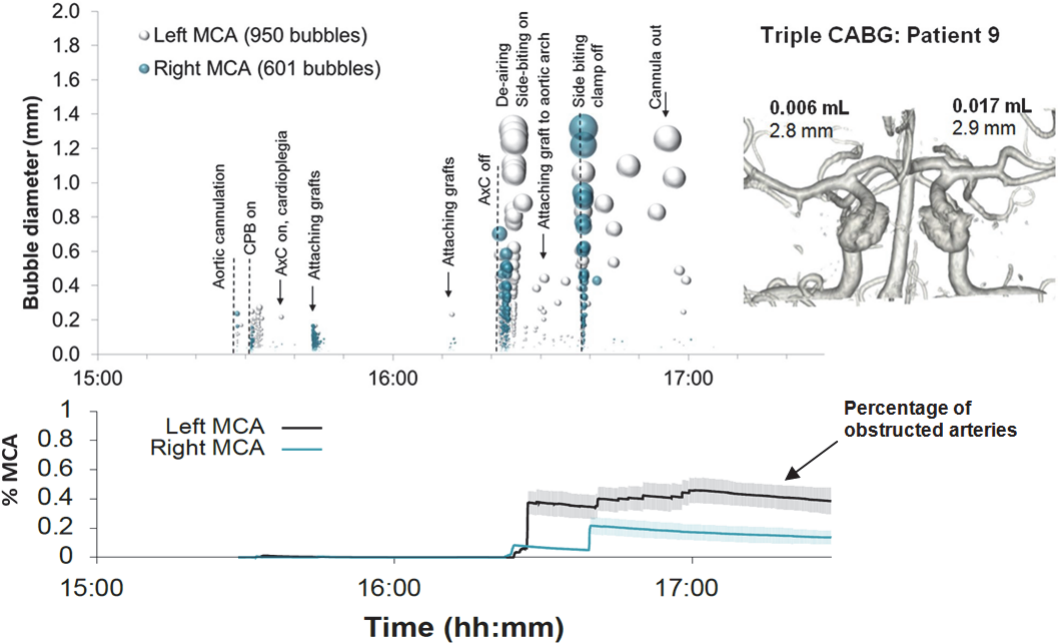
Timing and diameters of bubbles in a 76 year old patient during Triple CABG. Markers denote individual embolic events where the y-axis and marker size indicate estimated bubble diameter. The lower panel displays the predicted number of blocked MCA end arterioles estimated by Monte-Carlo simulation. The shaded regions highlight 95% confidence intervals, based on uncertainty in bubble diameter and variations in outcome between simulations. The inset shows a 3D reconstruction of the circle of Willis labelled with estimated total volume of air and MCA diameters.

**Fig 5:**
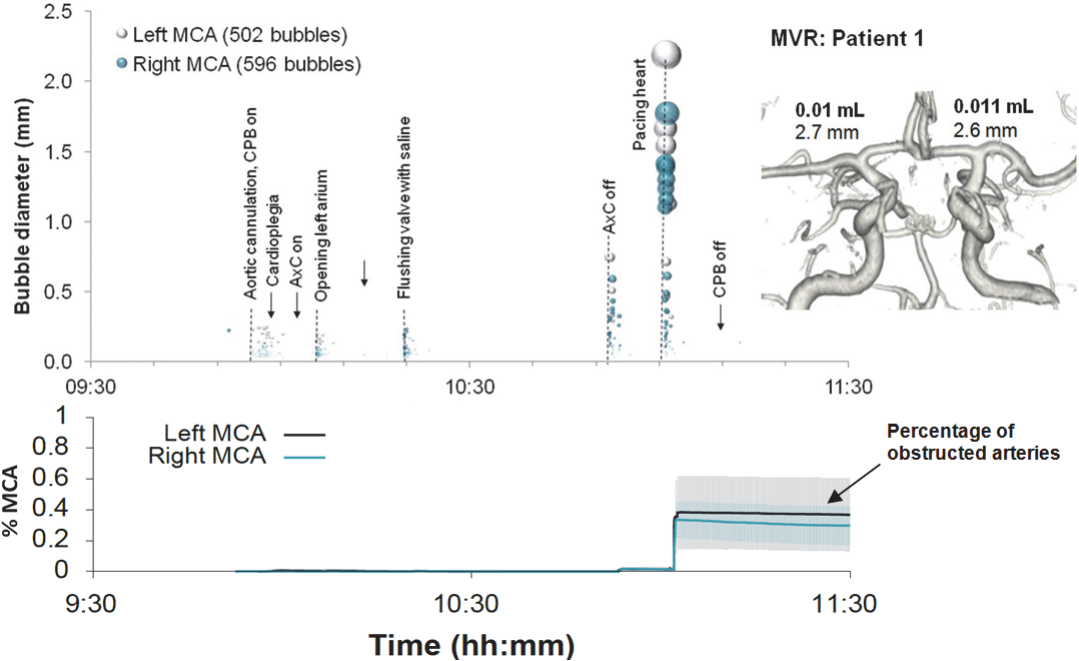
Timing and diameters of bubbles in a 71 year old patient during combined MVR and CABG. Markers denote individual embolic events where the y-axis and marker size indicates estimated bubble diameter. The lower panel displays the predicted number of blocked end arterioles obtained by Monte-Carlo simulation. The shaded regions highlight 95% confidence intervals, based on uncertainty in bubble diameter and variations in outcome between simulations. The inset shows a 3D reconstruction of the circle of Willis labelled with estimated total volume of air and MCA diameters.

**Fig 6:**
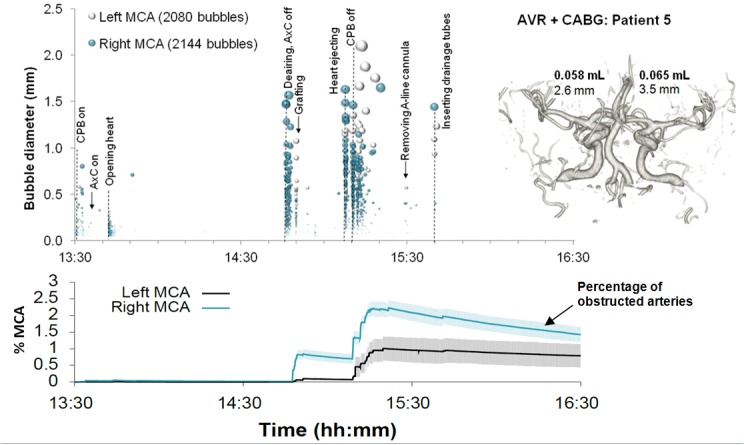
Timing and diameters of bubbles in a 55 year old patient during combined AVR and CABG. Markers denote individual embolic events where the y-axis and marker size indicates estimated bubble diameter. The lower panel displays the predicted number of blocked end arterioles obtained by Monte-Carlo simulation. The shaded regions highlight 95% confidence intervals, based on uncertainty in bubble diameter and variations in outcome between simulations. The inset shows a 3D reconstruction of the circle of Willis labelled with estimated total volume of air and MCA diameters.

Examples of detailed plots illustrating the timing and estimated sizes of bubbles in individual patients are presented in Figs 3–6. Each marker in the upper panel represents an individual embolic event. The y-axis and marker width shows estimated bubble diameter. The x-axis displays time during surgery. Closer examination of the timing of embolic signals shows that events during surgery generate distinct showers of emboli seen as vertical stripes in the plot. Showers typically occurred in left and right MCAs simultaneously and contain a broad distribution of bubble sizes. Restarting the heart following removal of the aortic cross-clamp consistently generated dense showers containing larger bubbles than during bypass. In four patients there were sections of the recording where embolic signals became too dense to analyse individually. We were unable to include these in our simulations but the durations of these 'curtains' of emboli are listed in Table 1.

By manually analysing data from the entire procedure, and maintaining detailed transcripts, we were able to pin-point potential sources of emboli showers (see labels in the upper panels of Figs 3–6). Light showers of bubbles were observed to coincide with the introduction and removal of cannulas, handling the heart, stitching grafts, and injections. Heavy showers were most often associated with the commencement of CPB, application and removal of the aortic cross-clamp (AxC), and restarting the heart during weaning from bypass. Nearly all patients experienced showers of emboli during commencement of bypass and following removal of the AxC.

Nearly three quarters of bubbles (73%) occurred prior to removal of the AxC during CPB, with an estimated median bubble diameter of 28 μm (IQR: 17 to 51 μm). Bubbles arising during CPB were numerous but considerably smaller than those entering the brain during the later stages of the surgery. The 28 μm median diameter of bubbles detected during CPB is consistent with the use of a 38 μm filter positioned in the aortic line of the CPB circuit.

Although the later stages of surgery, following removal of the AxC, contributed only 27% of embolic signals, these tended to include larger bubbles than during CPB, with a median bubble diameter of 72 μm (IQR: 28 to 202 μm). The majority of larger bubbles were observed as the heart began to eject following removal of the AxC. The largest detected bubbles (> 1 mm) reached diameters approaching the diameter of the MCA (e.g. ~3.5 mm in patient 5).

In individual patients, the total volume of air entering the MCAs during surgery varied from virtually no air at all [0.0002 mL (patient 8)] to a maximum of 0.12 mL (patient 5), Table 1. Patient specific Monte-Carlo estimates for the instantaneous percentage of obstructed end arterioles, with 95% Confidence Intervals, are plotted as the solid line in the lower panels of Figs 3–6. Our simulations suggest that small showers of filtered (<38 μm) bubbles occurring during bypass do not impair cerebral blood flow. The greatest predicted threats to perfusion estimated by our simulations were associated with unfiltered macrobubbles detected following removal of the AxC. However, showers of large emboli following removal of the AxC still only affected a small (<2.2%) percentage of the model vasculature.

## Discussion

This study represents a detailed survey of the backscatter intensities of Doppler embolic signals observed during cardiac surgery and estimates the likely size distribution of bubbles and volume of air entering the middle cerebral arteries. We confirm that the majority (~85%) of bubbles entering the cerebral circulation are less than 100 μm in diameter. The number and dimensions of air emboli revealed by our analysis is broadly consistent with previous autopsy studies by Moody *et al*., which revealed numerous small capillary arteriolar dilatations post-operatively.[15] Moody and colleagues studied 100 μm thick slices of the basal ganglia of patients who died within one week of surgery and observed numerous empty ~40 μm dilatations, with a density of approximately 40 dilatations per cm^2^ of tissue. The median diameter of air emboli estimated by our analysis of 33 μm (IQR: 18–71) is similar to Moody’s findings.

A limitation of our study was the difficulty in analysing heavy showers of emboli. During these 'curtains' of emboli some signals could not be analysed and the number of emboli, and volume of air received, would therefore have been underestimated; see Table 1 for curtain durations. This impacted mainly valve and combined procedure patients, where showers were particularly heavy. Based on these limitations, the numbers of emboli reported in our study for patients 1, 5, 6 and 9 should be considered a conservative estimate.

Our findings demonstrate that up to 0.12 mL of air can enter the MCA territories during surgery, Table 1. Although this volume of air is lower than the total volume of microbubbles found to generate acute cerebral injury in animal studies [6], as the dissolve time for an individual bubble depends crucially on surface area, and multiple bubbles have potential to coalesce, the potential for more subtle localised injuries cannot be completely discounted.

The time taken for an air embolus to completely dissolve, and proportion of the vascular tree that becomes obstructed, are both sensitively dependent on bubble size [1]; bubbles less than 38 μm clear within seconds, whereas a 1 mm bubble is thought to take over 5 hours, Fig 2(B). To better understand the relationship between vascular obstruction and bubble size relative to the dimensions of the arterial tree we used a Monte-Carlo simulation of gas bubbles moving through a model MCA vasculature.[9,13,14] Based on the results of these simulations, showers of bubbles with diameters less than ~38 μms did not generate any significant obstruction. Showers of larger (>100 μm) macrobubbles following removal of the aortic-cross clamp and during weaning from bypass were predicted to affect up to 2.2% of the model vasculature for several hours. However, a limitation of our model is the absence of a mechanism for allowing multiple small bubbles to coalesce. Large bubbles pose a much greater threat to blood flow because they take longer to dissolve and become lodged higher up in the arterial tree. If our simple model can be considered to reflect true levels of microvascular obstruction and rates of embolus clearance, the greatest threat to cerebrovascular perfusion is predicted to occur following removal of the AxC during weaning from bypass. A more sophisticated model would be required to model physiological responses associated with progression from obstruction to cerebral ischaemia by modeling oxygen diffusion in the capillary mesh, cerebral autoregulation, inflammation, and by including a detailed knowledge of the cerebrovasculature.

One of the major limitations of our study is that we have focused entirely on the impact of air emboli. Our current study is therefore limited to conclusions regarding the sizes of bubbles and overlooks the potential contribution to microvascular obstruction of solid particles. The presence of solid emboli would not affect our findings, as these would have been misclassified as very small bubbles, but would provide a significant additional embolic burden, which is not considered in the present study. To predict the impact of both solid and gaseous emboli, would require advances in distinguishing solid and gas emboli based on transcranial Doppler ultrasound.

Our study emphasises the importance of bubble size on the potential for air embolism and ischaemic damage to the brain. We implement a method for estimating bubble size based on the analysis of intra-operative transcranial Doppler ultrasound recordings. As large bubbles, containing a significant volume of air entering the circulation, are more clinically relevant than benign microbubbles, which pass harmlessly through the circulation, the ability to identify large macrobubbles intra-operatively through analysis of Doppler recordings could provide a valuable clinical monitoring tool during cardiovascular interventions. In future work we plan to use this technique, in combination with neuropsychological testing and MRI, to examine whether the presence of large macrobubbles, or high accumulated volume of air entering the circulation, provides an explanation for new brain injuries observed following heart surgery.
